# Changing muscle function with sustained glial derived neurotrophic factor treatment of rabbit extraocular muscle

**DOI:** 10.1371/journal.pone.0202861

**Published:** 2018-08-24

**Authors:** Krysta R. Fitzpatrick, Anja Cucak, Linda K. McLoon

**Affiliations:** 1 Department of Ophthalmology and Visual Neurosciences, University of Minnesota, Minneapolis, Minnesota, United States of America; 2 Department of Ophthalmology and Visual Neurosciences and Department of Neuroscience, University of Minnesota, Minneapolis, Minnesota, United States of America; Faculty of Medicine, Cairo University, EGYPT

## Abstract

Recent microarray and RNAseq experiments provided evidence that glial derived neurotrophic factor (GDNF) levels were decreased in extraocular muscles from human strabismic subjects compared to age-matched controls. We assessed the effect of sustained GDNF treatment of the superior rectus muscles of rabbits on their physiological and morphological characteristics, and these were compared to naïve control muscles. Superior rectus muscles of rabbits were implanted with a sustained release pellet of GDNF to deliver 2μg/day, with the contralateral side receiving a placebo pellet. After one month, the muscles were assessed using *in vitro* physiological methods. The muscles were examined histologically for alteration in fiber size, myosin expression patterns, neuromuscular junction size, and stem cell numbers and compared to age-matched naïve control muscles. GDNF resulted in decreased force generation, which was also seen on the untreated contralateral superior rectus muscles. Muscle relaxation times were increased in the GDNF treated muscles. Myofiber mean cross-sectional areas were increased after the GDNF treatment, but there was a compensatory increase in expression of developmental, neonatal, and slow tonic myosin heavy chain isoforms. In addition, in the GDNF treated muscles there was a large increase in Pitx2-positive myogenic precursor cells. One month of GDNF resulted in significant extraocular muscle adaptation. These changes are interesting relative to the decreased levels of GDNF in the muscles from subjects with strabismus and preliminary data in infant non-human primates where sustained GDNF treatment produced a strabismus. These data support the view that GDNF has the potential for improving eye alignment in subjects with strabismus.

## Introduction

A number of studies in chick and adult rabbit have demonstrated that extraocular muscle (EOM) structure and function can be significantly modified by exposure to selected neurotrophic factors or their inhibitors [1–5]. These initial studies were extended using the non-human primate, where sustained release of insulin growth factor -1 (IGF-1) was able to produce a strabismus in infant monkeys [6] as well as improve eye alignment in an adult monkey with a sensory-induced strabismus [7]. A large number of neurotrophic factors are expressed in the extraocular muscles and motor neurons, but not all factors tested thus far using sustained treatment were similarly effective in producing strabismus [6,8]. For example, 3 months of sustained release of brain derived neurotrophic factor (BDNF) in infant monkey EOM did not produce a strabismus, although it did result in significant alterations in myofiber types within the treated lateral rectus muscles [8].

Recent studies used DNA microarray and RNAseq analyses to compare EOM removed during strabismus surgery in adults with EOM from age-matched controls [9,10]. These studies showed that the EOM from the strabismic subjects not only had increased expression of IGF binding proteins 5 and 6, both of which inhibit IGF-1 function, but also showed evidence of a significant down-regulation of both ciliary neurotrophic factor (CNTF) and glial derived neurotrophic factor (GDNF) [9,10]. Collectively, these studies link alterations in levels of specific neurotrophic factors to the development and/or maintenance of strabismus. They further support the hypothesis that modulating neurotrophic factor levels with exogenous treatment has great potential for the treatment of strabismus.

GDNF plays a number of diverse roles in skeletal muscle development and function. It was demonstrated to be a potent survival factor for motor neurons during development [11]. More specifically, it was a critical factor in the early developmental survival of oculomotor neurons [12,13]. Exogenous GDNF treatment was shown to prevent death of oculomotor, trochlear, and abducens neurons after axotomy in neonatal rats [14]. Using a chick model, injection of GDNF into the orbit resulted in a shorter contraction profile—essentially making the eye movements more rapid [15]. Together, these studies suggest that alterations in GDNF signaling play a role in the etiology of childhood onset strabismus. Our preliminary study showed that GDNF treatment of infant monkey EOM resulted in the development of strabismus [16]. To understand the physiological and structural changes produced by GDNF treatment, adult rabbit superior rectus muscles were implanted with a pellet that provided sustained release of GDNF for a period of one month. *In vitro* physiologic properties were examined in the GDNF-treated superior rectus muscles, and these data were compared to the contralateral untreated superior rectus muscle as well as to naïve control superior rectus muscles. These muscles were then examined histologically and morphometrically for changes in myofiber size, innervation density, myosin heavy chain isoform (MyHC) alterations, and changes to myogenic precursor cell populations. This study sheds light on potential mechanisms by which alteration in GDNF signaling might produce strabismus.

## Methods

Adult New Zealand white rabbits were obtained from Bakkom Rabbitry (Viroqua, WI) and maintained by the animal facility at the University of Minnesota. All experiments were approved by the Institutional Animal Care and Use Committee at the University of Minnesota. All procedures were in compliance with the standards provided by the National Institutes of Health and the animal care and use standards set by the Association for Research in Vision and Ophthalmology.

Five rabbits were anesthetized with 0.6ml/kg mixture of ketamine:xylazine (4:3; 100mg/ml and 20mg/ml, respectively). Under sterile conditions, a small incision was made in the conjunctiva, and the superior rectus muscle was identified and isolated on a muscle hook. Pellets were commercially prepared for us, and tested for their release profile prior to shipment. We have used this sustained release method in previous studies and saw clear effects over time, confirming the efficacy of the sustained release [6–8]. One pellet containing GDNF (R and D Systems, Minneapolis, MN), which released 2μg of the factor per day (Innovative Research of America, Sarasota, FL), was placed between the sclera and the muscle as far posteriorly as possible, minimally disturbing the superior rectus muscle. The conjunctiva was then closed. A placebo pellet was similarly placed on the contralateral superior rectus muscle. GDNF-containing pellet placement was randomized for each rabbit. Tobradex ointment was placed in the conjunctival cul-de-sac at the end of surgery. These were compared to 5 control rabbits that did not receive neurotrophic factor treatment.

After one month, the rabbits were euthanized, and the superior rectus muscles were removed from the sclera to the orbital apex and placed in oxygenated Ringer’s solution. Both ends of the muscles were tied with 4–0 suture, and the suture was used to attach the muscles to a force transducer via a lever arm and a stable bar in order to determine the *in vitro* contraction profile of each muscle (Aurora Scientific, Aurora, Ontario, Canada) using our standard protocol [1]. Briefly, the muscles were placed in a 32°C bath containing oxygenated Ringer’s solution and stimulated globally using two flanking electrodes. Optimal preload was determined, and after a 15 minute rest, the muscles were sequentially stimulated at a single twitch pulse, double twitch pulse, and a triple twitch pulse, followed by stimulation at 10Hz, 20Hz, 40Hz, 100Hz, 150Hz, and 200Hz, with 2 minutes rest between each stimulation. The twitch and tetanic force profiles were determined from these measurements. These included peak force, defined as the maximal force at a specific stimulation frequency in grams, time to 50% and 100% relaxation in seconds, peak relaxation rate in grams/second (dF/dt), time to maximum force in seconds, and peak contraction rate in grams/second (dF/dt). After physiological assessment, the muscles were weighed and their length determined in order to convert force from grams to milliNewtons/centimeter^2^ (mN/cm^2^) [4].

After the physiological analysis, the superior rectus muscles were embedded in tragacanth gum and frozen in 2-methylbutane chilled in liquid nitrogen. Sections were cut on a cryostat at 12μm, and the slides were stored at -30°C until stained. One series of sections were stained with hematoxylin and eosin to use for analysis of mean myofiber cross-sectional area. Myofiber areas were determined by manual tracing using the Bioquant Image Analysis System (Bioquant, Nashville, TN). Between 200 and 300 myofibers were measured in both the orbital and global layers both at the middle region and towards the tendon region. Three slides were analyzed in each region, and the means from each muscle were determined and used to calculate the overall mean myofiber areas for the GDNF-treated superior rectus muscles, the placebo-treated superior rectus, and a set of naïve control superior rectus muscles.

Another series of sections were immunostained for a panel of myosin heavy chain isoforms (MyHC) including slow twitch (1:100, Hybridoma Bank, Ames, Iowa), slow tonic (1:50, Hybridoma Bank), developmental (1:20, Leica, Buffalo Grove, IL), and neonatal (1:20, Hybridoma Bank) diluted in antibody buffer containing phosphate buffered saline (PBS) and 0.1% triton X-100. After a rinse in PBS, the sections were incubated at room temperature in the appropriate normal serum, followed by incubation with the primary antibody for one hour, and a rinse in PBS. The slides were incubated in a secondary antibody conjugated to horseradish peroxidase (Vector Labs., Burlingame, CA), and processed by incubation in diaminobenzidine containing heavy metals. The percent of myofibers positive for each of these MyHC isoforms was determined for both the orbital and global layers for three slides in both the middle region and towards the tendon end, with 200–300 fibers analyzed per cross-section. The means for both the treated and contralateral superior rectus muscles from each animal were calculated, and these were averaged and compared to naïve control superior rectus muscles to determine the overall change in the percent of myofibers expressing an individual MyHC isoform.

Neuromuscular junction density and size were measured for these muscles using Bioquant software. Sections were incubated as described above using an antibody to slow twitch MyHC (Hybridoma Bank), rinsed in PBS, blocked in 20% normal serum, followed by an incubation with anti-mouse IgG conjugated to Cy3 (1:500, Jackson ImmunoResearch Labs., West Grove, PA). After a PBS wash, sections were incubated at room temperature for 1 hour in alpha-bungarotoxin conjugated to Alexafluor 488 (1:500, Invitrogen, Carlsbad, CA). The sections were rinsed in PBS and mounted with Vectashield (Vector Labs.). To determine neuromuscular junction density, all the neuromuscular junctions in entire cross-sections from three slides each in both the middle and tendon regions of each of the 3 groups of superior rectus muscles were counted: naïve control, GDNF-treated, and muscles contralateral to the treatment. After the neuromuscular junction counts were made, the entire cross-section was traced. This allowed calculation of the number of neuromuscular junctions per cross-sectional area. These were averaged for each region of a given muscle, and these averages were used to determine statistical significance. To determine mean neuromuscular junction size, lengths of individual neuromuscular junctions were measured, as well as the perimeter of the fibers on which they were found. Similarly areas of the individual neuromuscular junctions were determined, as well as their corresponding myofiber areas. These measurements allowed calculation of mean length, mean area, mean length as a percent of fiber perimeter, and mean area as a percent of fiber area. All three groups of superior rectus muscles were analyzed.

To assess the effect of one month GDNF treatment on myogenic precursor cell populations, superior rectus muscle cross-sections in the middle region and toward the tendon end were processed for the immunohistochemical localization of Pax7 and Pitx2. For Pax7 staining, sections were first quenched in 0.3% hydrogen peroxide, rinsed in PBS, fixed in cold acetone, blocked in 20% normal serum, further blocked using the avidin/biotin blocking kit (Vector Labs.), and followed by incubation in a primary antibody to Pax7 (1:500, Hybridoma Bank) diluted in antibody buffer. The sections were rinsed in PBS, incubated in secondary antibody using the HRP method (Vector Labs.), and processed by incubation in diaminobenzidine containing heavy metals. Pitx2 was visualized using immunofluorescence. After incubation in 20% normal serum, the sections were incubated in an antibody against Pitx2 (1:400, abcam, Cambridge, MA) for 1 hour at room temperature. After a PBS rinse and a block in 20% normal serum, the sections were incubated in anti-mouse IgG conjugated to Alexafluor 488 (1:100, Jackson ImmunoResearch). Sections were blocked in 10% mouse serum for 1 hour at room temperature, rinsed in PBS, and incubated overnight at 4°C with AffiniPure Fab fragment goat anti-mouse IgG (1:100, Jackson ImmunoResearch). After a rinse in PBS, the sections were blocked in 20% normal serum, followed by an incubation in an antibody to dystrophin (1:200, Sigma, St. Louis, MO), rinsed in PBS, blocked in 20% normal serum, and incubated in anti-mouse IgG conjugated to Cy3 (1:500; Jackson ImmunoResearch). For analysis, in each microscopic field, positive cells and fiber number were determined for Pax7-positive and Pitx2-positive cells. Pitx2 positive nuclei inside the dystrophin ring, which are myonuclei, and outside the dystrophin ring, which are myogenic precursor cells, were counted. This quantification was performed for the orbital and global layers in the distal half of the muscles, with 6 slides counted for each region and muscle. The final statistical analyses were performed using averaged counts from each rabbit including both regions.

Statistical analysis was performed using GraphPad Prism software (Graphpad, San Diego, CA). Data between groups were analyzed for significance using an ANOVA followed by Tukey’s multiple comparison tests. Statistical significance was defined as p<0.05.

## Results

### *In vitro* physiology

**Fig 1:**
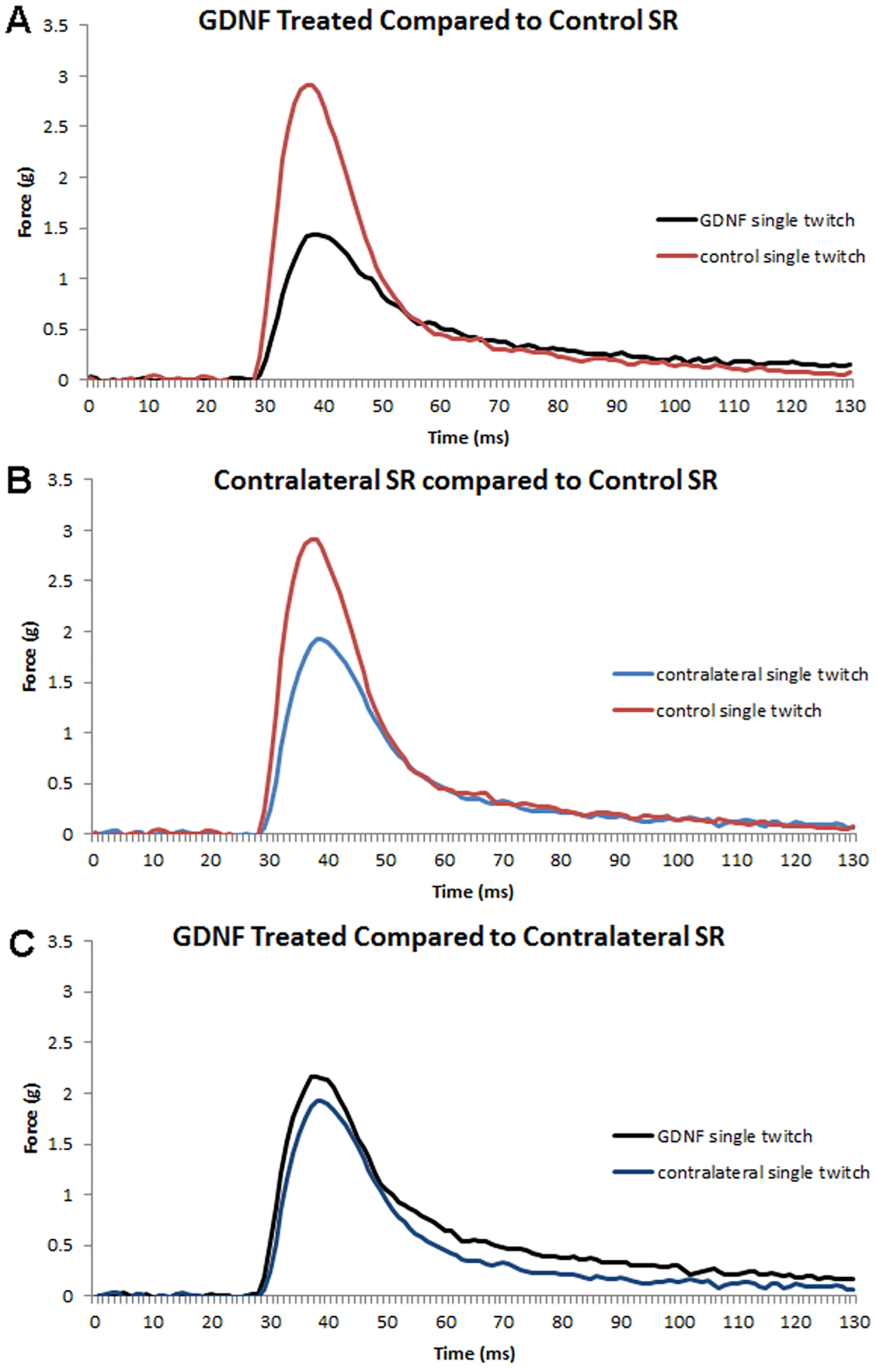
Graph of the response to stimulation of superior rectus muscles *in vitro* at twitch frequency. Red lines indicate naïve control muscles, black lines indicate GDNF-treated muscles, and blue lines indicate muscles contralateral to the treated muscles.

**Fig 2:**
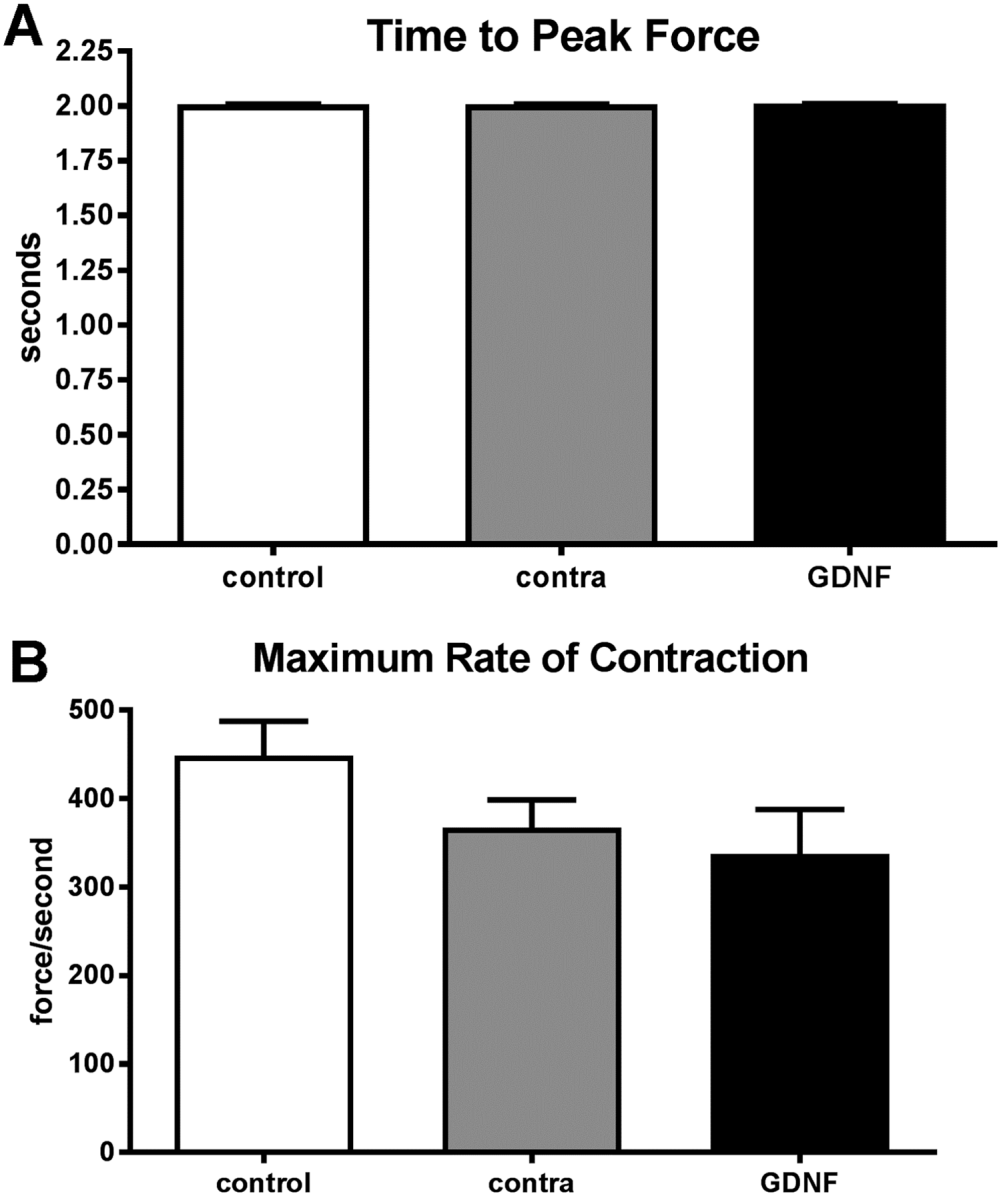
A. Time to peak force in seconds at twitch stimulation frequency of naïve control (white bar), muscle contralateral to the treatment (gray bar), and the GDNF-treated muscles (black bar). B. Maximum rate of contraction at twitch stimulation frequency of naïve control (white bar), muscle contralateral to the treatment (gray bar), and the GDNF-treated muscles (black bar). * indicates significant difference from naïve control muscles.

One month of GDNF treatment resulted in significant changes to a number of physiological properties of the treated superior rectus muscles compared to age-matched control muscles. Both the GDNF-treated muscles and the superior rectus muscles contralateral to the treated muscles had altered contraction profiles compared to the naïve control muscles (Fig 1). There were no significant differences between control, GDNF-treated, or contralateral superior rectus muscles in the time to peak force after twitch stimulation (Fig 2A). However, at the higher stimulation frequencies, the time to peak force was significantly greater for both the GDNF-treated muscles and the muscles contralateral to the treatment compared to the control muscles (data not shown). At the twitch frequency, the maximum rate of contraction was not significantly different from controls, despite the contralateral and GDNF-treated superior rectus muscles being 20% and 28.4% slower than the control muscles (Fig 2B).

**Fig 3:**
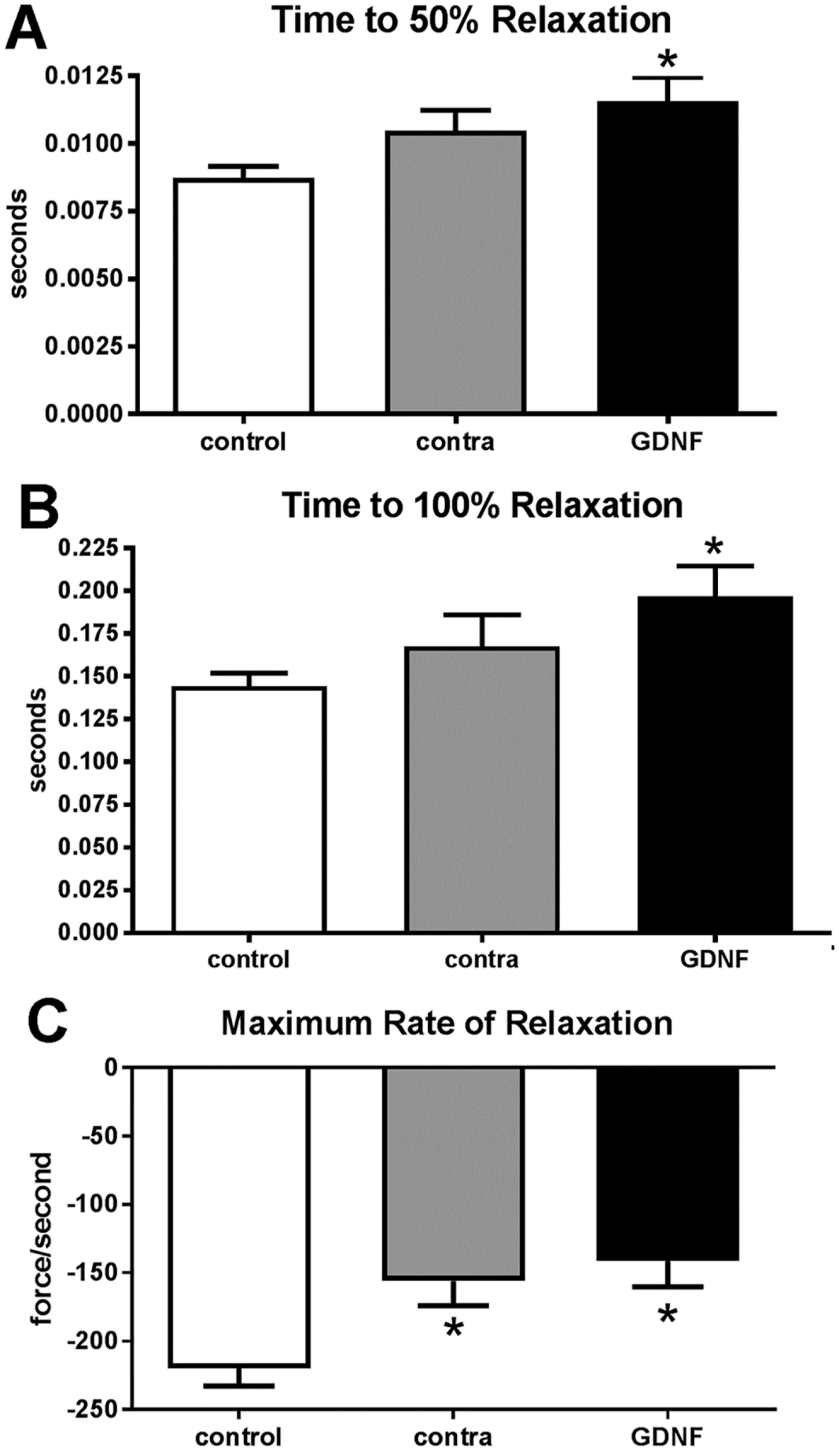
At twitch stimulation frequency graphs indicate (A) time to 50% relaxation, (B) time to 100% relaxation, and (C) maximum rate of relaxation of naïve control muscles (white bar), muscles contralateral to the treatment (gray bar), and the GDNF-treated muscles (black bar). * indicates significant difference from naïve control muscles.

At the twitch stimulation frequency, the times to 50% and 100% relaxation were significantly lengthened in the GDNF-treated muscles compared to the naïve control muscles (Fig 3A and 3B). Specifically, the time to 50% relaxation was 28% slower in the GDNF-treated muscles compared to controls. While not significantly different, the time to 50% relaxation for the muscles contralateral to the treatment was 18% slower than the controls. Similarly, at twitch stimulation the time to 100% relaxation was significantly slower in the GDNF treated muscles, 30.6% slower than the naïve control muscles. The time to 100% relaxation for the contralateral muscles was intermediate between the control and GDNF-treated superior rectus muscles. Although not significantly different, the time to 100% relaxation for the muscles contralateral to the treated ones were 14.9% slower than the naïve control muscles (Fig 3B). Both the GDNF-treated and the contralateral superior rectus muscles displayed a significantly prolonged rate of relaxation, with a 43.4% and 34% slower rate of relaxation than the naïve control muscles, respectively (Fig 3C).

**Fig 4:**
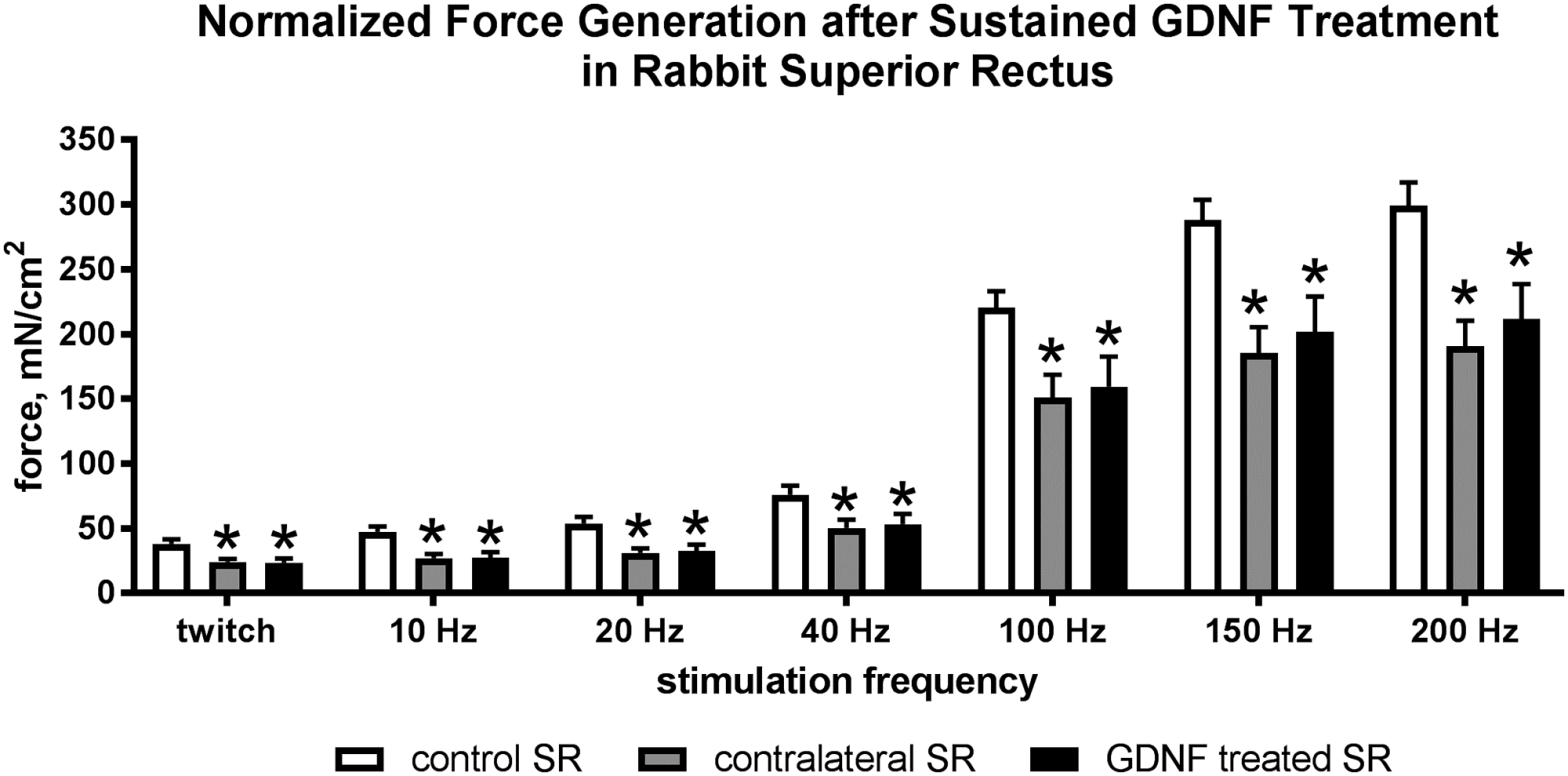
Force generation in mN/cm^2^ after twitch, 10Hz, 20Hz, 40Hz, 100Hz, 150 Hz and 200 Hz stimulation of naïve control muscles (white bars), muscles contralateral to the treatment (gray bars), and GDNF-treated muscles (black bars). * indicates significantly different from naïve control muscles.

Force generation is acquired in grams and converted to mN/cm^2^ based on muscle weight and length as we have done previously [4]. When force (in grams) was examined, there were no significant differences between force generated from the GDNF-treated muscles compared to either the contralateral superior rectus muscles or the naïve controls (data not shown). However, when converted to force relative to muscle length and weight, in mN/cm^2^, force generation was significantly lower at all stimulation frequencies for the GDNF treated muscles, with decreases of 38.2%, 42.2%, 39.5%, 29.8%, 27.8%, 29.9%, and 29.1%. Interestingly, the contralateral superior rectus muscles also produced significantly less force than naïve control rabbit superior rectus muscles were decreased from naïve control muscles by 36.8%, 42.9%, 42.4%, 33.7%, 31.7%, 35.7%, and 36.3%. These were similar to the forces generated on the GDNF-treated side (Fig 4).

### Mean myofiber cross-sectional areas

**Fig 5:**
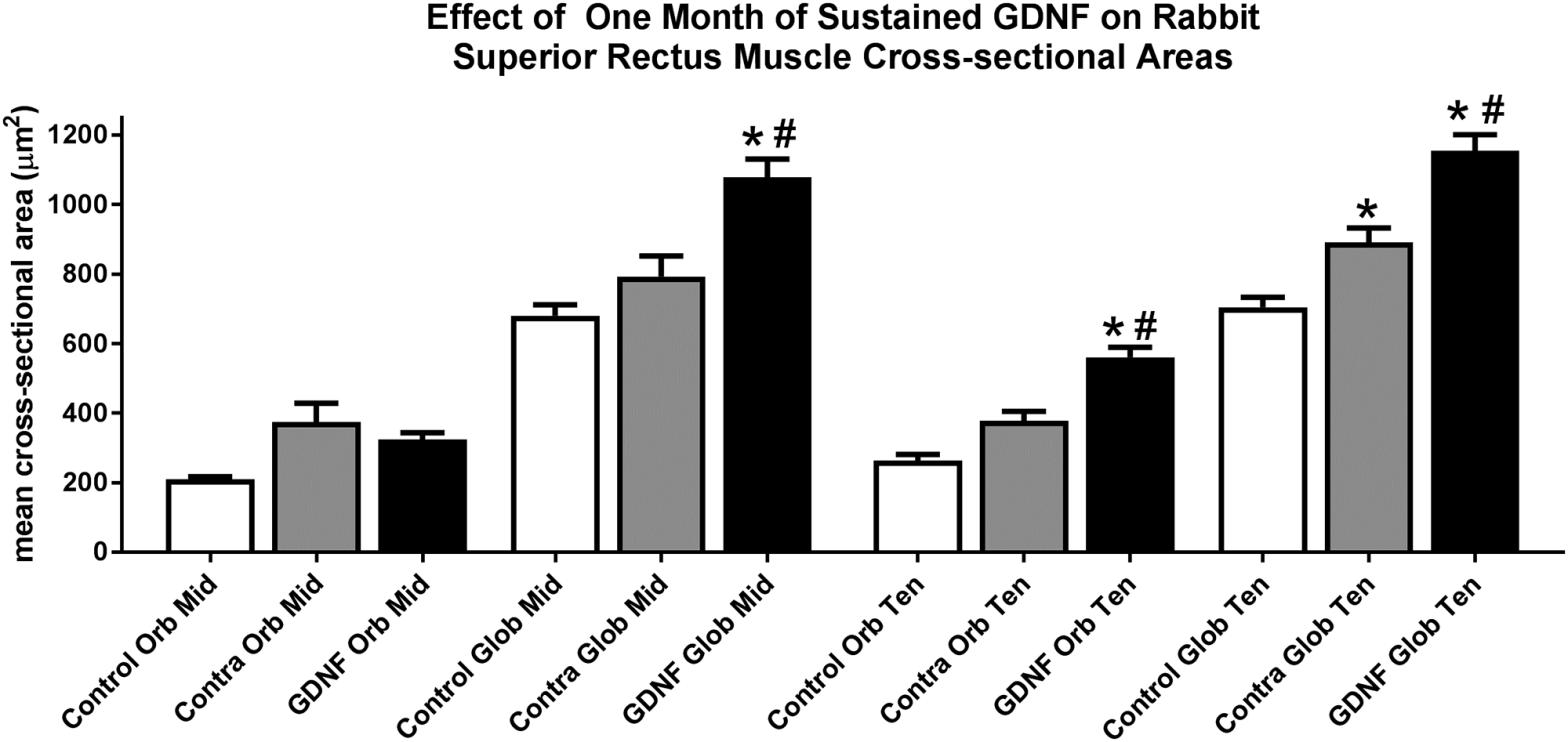
Effect of sustained GDNF treatment on mean cross-sectional areas (μm^2^) from naïve control muscles (white bar), muscles contralateral to the treatment (gray bar), and the GDNF-treated muscles (black bar). mid: middle region; ten: tendon region; orb: orbital; glob: global. * indicates significant difference from naïve control muscles. # indicates significant difference from the muscle contralateral to the treated muscle.

The mean cross-sectional areas of the GDNF-treated superior rectus muscles were compared to the contralateral superior rectus muscles and those from untreated naïve controls in both the orbital and global layers in the middle of the muscles and towards the tendon end (Fig 5). There were no significant differences seen in the orbital layers of the middle region between these three groups of muscles. In the global layer of the middle region, the GDNF-treated superior rectus mean cross-sectional areas were significantly larger than the naïve control muscles, a difference of 58.5%. These muscles were also significantly larger than the superior rectus muscles contralateral to the GDNF treatment, a difference of 36%. In the orbital layer of the muscles toward the tendon end, the GDNF-treated myofibers were significantly larger than the mean areas in naïve control muscles, an increase of 112.7%. The muscle fibers in the orbital layer of the muscles contralateral to the treatment were not significantly different from the age-matched controls, albeit 43.3% larger than mean area of the naïve control orbital layer myofibers. The GDNF-treated orbital layer myofibers towards the tendon region were also significantly larger than the muscles contralateral to the GDNF treatment, a difference of 48.4%. Similarly, in the global layer of the superior rectus muscles closer to the tendon region, the mean cross-sectional areas of both the muscles contralateral to treatment and the GDNF-treated muscles were significantly larger than the mean areas in naïve control muscles, with differences of 26.4% and 63.9% respectively. The GDNF-treated muscles in the global layer near the tendon were also significantly larger than the muscles contralateral to the GDNF treatment, a difference of 29.6%.

### Myosin heavy chain isoform (MyHC) expression changes

**Fig 6:**
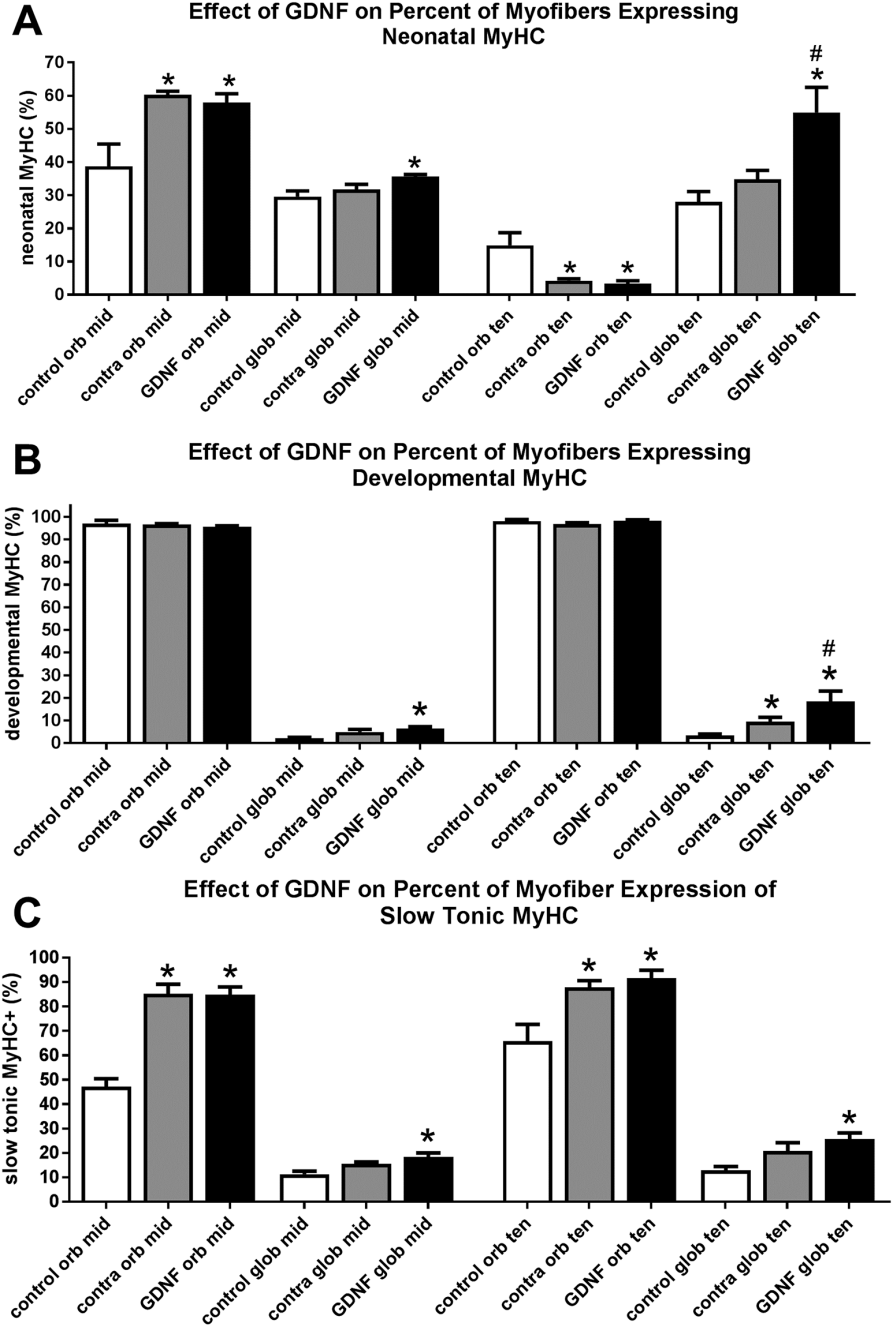
Effect of sustained GDNF treatment on the percent of myofibers expressing the neonatal myosin heavy chain (MyHC) isoform (A) the developmental MyHC isoform (B), and the slow tonic MyHC isoform (C). mid: middle region; ten: tendon region; orb: orbital; glob: global. * indicates significant difference from naïve control muscles. # indicates significant difference from the muscle contralateral to the treated muscle.

As shortening velocity is in part controlled by MyHC isoform expression [17–19], several MyHC isoforms were examined in these three groups of muscles. There were significant alterations in the percent of myofibers expressing neonatal MyHC (*MYH8*) (Fig 6A), developmental MyHC (*MYH3*) (Fig 6B), and slow tonic MyHC (*MYH7b*) (Fig 6C).

In the GDNF-treated muscles and the muscles contralateral to the GDNF treatment, there were significant increases in the percent of myofibers that expressed neonatal MyHC in the orbital layer in the middle region after treatment, with increases of 49.6% and 55.7% over naïve control muscles, respectively. There was a decrease in the percent of myofibers that expressed neonatal MyHC in the orbital layer in the tendon region; however, the overall percentage of neonatal positive fibers is low in this region. In the naïve control muscles, 14.9 ± 3.8% were neonatal MyHC positive, while the treated superior rectus muscle was 3.36 ± 0.86% positive and the contralateral muscles were 4.23 ± 0.6% positive. Significant increases were seen in the global layers of both the middle region and the tendon region of the GDNF-treated muscles, with increases of 20.4% and 96.2% respectively (Fig 6A). This is interesting in light of the correlation between levels of neonatal MyHC expression and decreased shortening velocity and force production seen in developing compared to mature muscles [20].

Developmental MyHC expression was unchanged from naïve control superior rectus muscles in the orbital layers in the middle and tendon regions (Fig 6B). The endplate zone was not included in our analyses, as this region does not express this MyHC isoform [21]. Developmental MyHC expression significantly increased in the GDNF-treated global layers compared to naïve control muscles in the middle and tendon regions (Fig 6B). The middle region of GDNF-treated muscles showed a significant 189% increase in number of positive fibers compared to naïve controls. The middle region of muscles contralateral to the treated superior rectus muscles showed a 122.7% increase in percent positive compared to naïve controls, but this difference was not significant (Fig 6B). In the region toward the tendon end, the GDNF-treated muscles had a 452.4% increase in percent of myofibers positive for developmental MyHC, and the contralateral side showed a 184.1% increase over naïve controls, both significantly elevated (Fig 6B).

While there were no significant differences in the percentages of myofibers expressing the slow twitch (*MYH7*) MyHC isoform (data not shown), there were significant increases in the percentage of orbital myofibers expressing slow tonic MyHC in GDNF-treated muscles and the muscles on the side contralateral to the treatment compared to naïve control muscles (Fig 6C). These represented 74.4% and 73.9% increases, respectively, in the middle region and 28.6% and 32.7% increases, respectively, in the tendon region. In the GDNF-treated muscles, there were 47.6% more slow tonic-positive global fibers in the middle region and 66.5% more in the tendon region, significantly more than in the naïve control muscles. The muscles on the side contralateral to the GDNF treatment in the middle and tendon regions had 31.4% and 47.1% more slow tonic-positive fibers than the naïve control muscles, respectively, but these differences were not significant. Other MyHC isoforms examined had minimal changes after the one month of sustained GDNF treatment compared to untreated naïve controls (data not shown).

### Neuromuscular junction analysis

**Fig 7:**
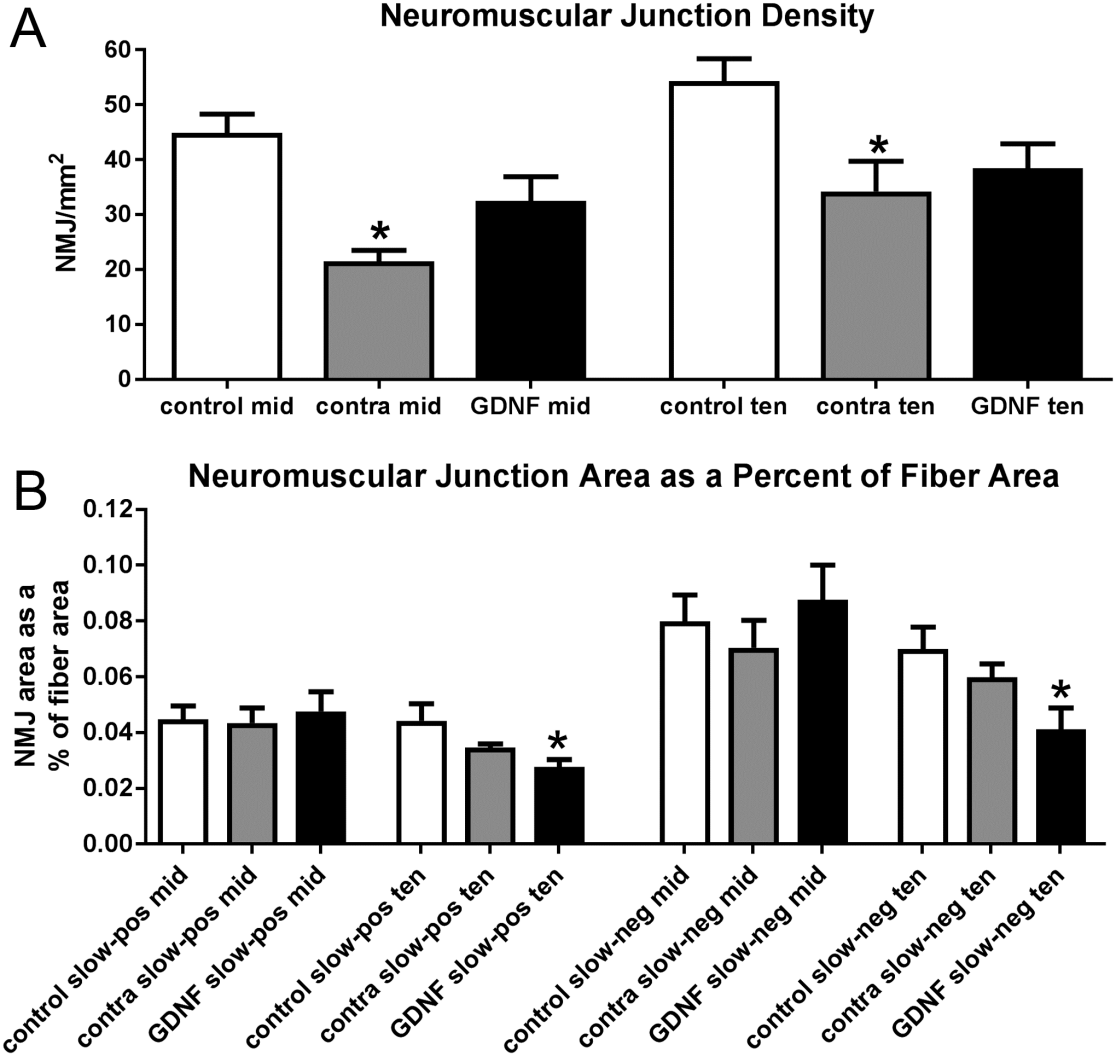
A. Neuromuscular junction density as a proportion of mm^2^ of muscle area. NMJ: neuromuscular junction; mid: middle region; ten: tendon region. * indicates significant difference from naïve control muscles. B. Neuromuscular junction area as a percent of fiber area was determined for both slow-positive and slow-negative myofibers in the middle region and towards the tendon end of naïve control, GDNF-treated, and contralateral untreated superior rectus muscles. NMJ: neuromuscular junction; mid: middle region; ten: tendon region; pos: slow-positive; neg: slow-negative. * indicates significant difference from naïve control muscles.

The increased mean myofiber area was surprising considering the decrease in force generation relative to muscle size in the GDNF-treated superior rectus muscles. The density of neuromuscular junctions was assessed as studies have suggested that GDNF can have a significant impact in both density and size of neuromuscular junctions [22–24]. Calculated as neuromuscular junctions per mm^2^ of muscle area, there was a significant decrease in the density of neuromuscular junctions in the superior rectus muscles contralateral to the GDNF-treated muscles compared to naïve control superior rectus muscles in both the middle region and towards the tendon end (Fig 7A), decreases of 70.5% and 45.43% respectively. The neuromuscular density of both the middle region and tendon end of the GDNF treated muscles was 31.7% and 34.3% less than the controls, respectively, but neither difference was statistically significant.

Neuromuscular junction areas were calculated as a percent of myofiber area for all three groups of muscles in the middle and towards the tendon end on both slow-positive and slow-negative myofibers. Interestingly, significant changes were seen in this ratio in the tendon region on both slow-positive and slow-negative GDNF-treated myofibers, which were 37.3% and 41.2% smaller than those on naïve control muscles (Fig 7B).

### Myogenic precursor populations

**Fig 8:**
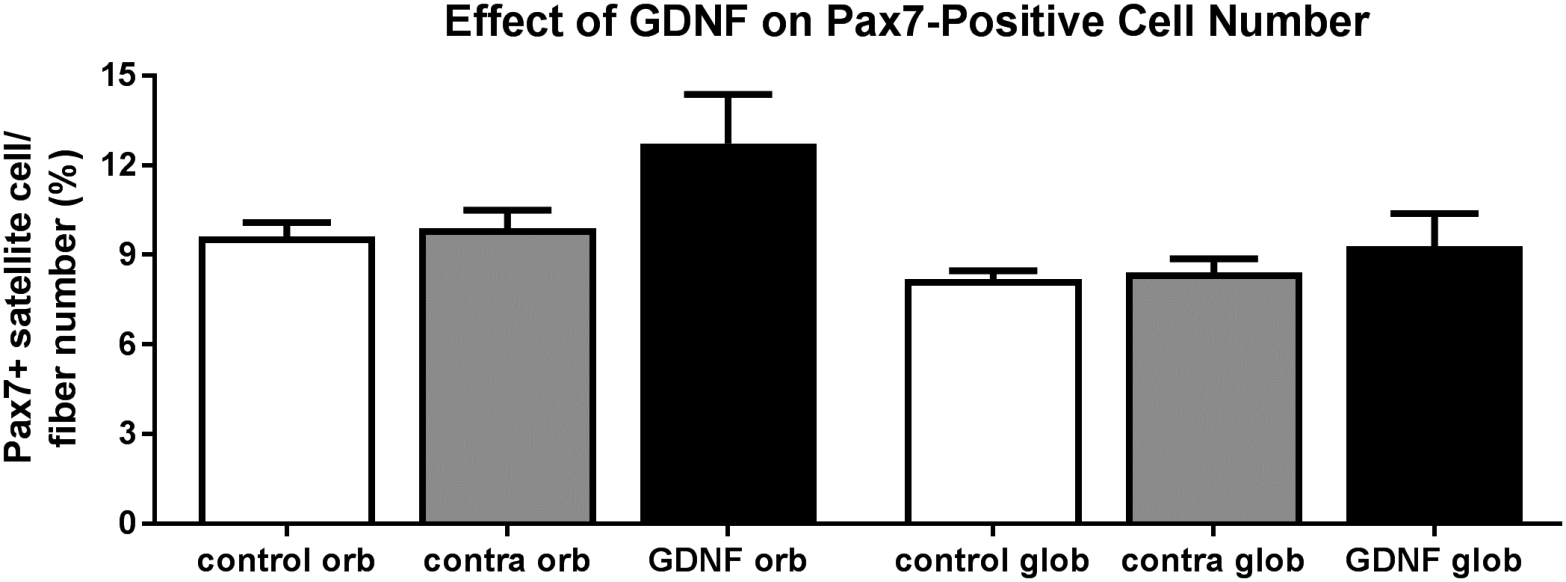
Effect of sustained GDNF treatment on Pax7-positive cells as a percent of fiber number. orb: orbital; glob: global.

One potential explanation for the increased cross-sectional area of the GDNF-treated muscles is that GDNF might foster a proliferative response in the muscle regenerative populations. Pax7 is a transcription factor expressed on quiescent satellite cells in all skeletal muscles and plays an important role in skeletal muscle growth, repair, and regeneration [25, 26], with elevated levels in extraocular muscles [27]. Analysis showed that there were no significant differences in the number of Pax7-positive satellite cells per myofiber number between any of the areas or regions (Fig 8).

**Fig 9:**
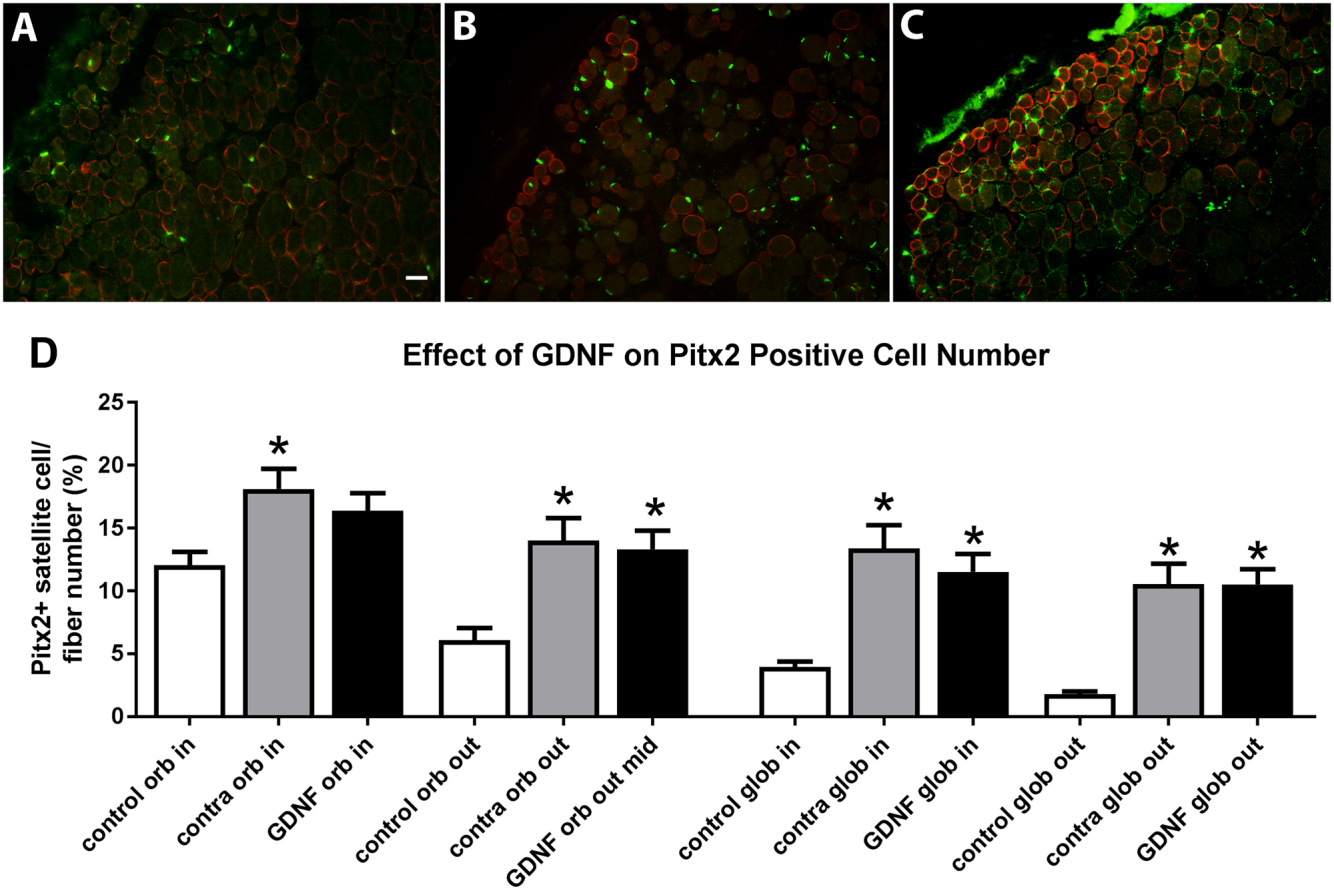
Photomicrographs of cross-sections immunostained for Pitx2 (green) and dystrophin (red) from (A) naïve control superior rectus muscle, (B) superior rectus muscle contralateral to a GDNF treated muscle, and (C) GDNF treated superior rectus muscle. (D) Pitx2 expression as a percent of fiber number in naïve control muscles, the muscle contralateral to GDNF treatment, and in the GDNF-treated muscles. Both Pitx2 myonuclei (in) and Pitx2 myogenic precursor cells (out) were analyzed in the orbital (orb) and global (glob) layers of the muscles. * indicates significant difference from naïve control muscles. Magnification bar is 20μm.

Extraocular muscles also contain a second population of myogenic precursor cells that is highly enriched compared to their levels in limb skeletal muscle, first identified by their expression of CD34 while negative for endothelial and hematopoietic markers [28], and further by their expression of the transcription factor Pitx2, and are highly enriched in the extraocular muscles [29]. In addition, there are large numbers of Pitx2-positive myonuclei in extraocular muscles. We hypothesize that these myonuclei represent the addition of Pitx2 precursor cells into existing fibers during the continuous EOM myofiber remodeling that is a normal process in adult EOM [29,30,31]. Examination of both Pitx2-positive myonuclei and Pitx2-positive myogenic precursor cells showed that both were significantly increased in the GDNF-treated superior rectus muscles and in the superior rectus muscles on the contralateral side to the treatment except for Pitx2-positive myonuclei in the orbital layer (p = 0.08) (Fig 9A–9D). Compared to naïve control superior rectus muscles, there was a 40.2% and 30.5% difference in Pitx2-positive myonuclei in the orbital layer and a significant 108.8% and 97.9% difference in the global layer in the muscle contralateral to treatment and the treated muscles, respectively. When myogenic precursor cells were analyzed, there was a significant 79% and 74.7% difference in the orbital layer and 141.2% and 141.7% difference in the global layer in the muscle contralateral to treatment and the treated muscles, respectively.

## Discussion

Treatment of rabbit superior rectus muscles unilaterally with sustained release GDNF resulted in significant changes to muscle contractile properties at twitch frequencies, specifically increased time to 50% and 100% relaxation, and a slower rate of relaxation. Twitch frequency responses showed an increased time to 50% and 100% relaxation. Additionally, at the higher stimulation frequencies, force generation, in mN/cm^2^, was significantly reduced in both the GDNF-treated muscles and the untreated contralateral muscles. These responses at different stimulation frequencies are interesting, as the GDNF resulted in a muscle that produced significantly less force than the naïve control muscles that also took longer to relax back to the pre-stimulated state.

Little is known about the effect of GDNF overexpression on these types of physiologic properties of adult skeletal muscle and more specifically adult EOM. Ocular motor neurons were shown to depend on GDNF retrogradely transported from the extraocular muscles to the motor neurons for their survival during development [12, 13]. GDNF also had neuroprotective effects after nerve injury in neonatal rats [14]. GDNF effects on muscle function were examined in developing chicks by blocking its function using an antibody against GDNF in combination with antibodies to insulin-like growth factor-1 and cardiotrophin-1 as part of an orbital injection of an antibody cocktail [2]. Subsequently, GDNF was injected into the chick orbital space, and in contrast to the present study, this treatment shortened contraction time but did not alter muscle force generation [15]. While direction injection is a viable way to test potential efficacy of a specific neurotrophic factor, the use of sustained release treatments allows for the potential that these changes might be longer lasting and therefore more efficacious. Our own studies have shown that maximal effects of treatment only occur with those of longer duration [1,3,6–8] It is interesting to note that in the present study alteration in measurable force relative to muscle size of the GDNF-treated superior rectus muscles was seen in both the treated muscles and in the muscles contralateral to the GDNF treatment. These initial studies in developing chick EOM used one or two orbital injections, rather than the one month of sustained delivery used in the present study. Thus the dose and treatment duration was markedly different between these two studies. Further work is needed to assess these differences. In particular, examination of the motor units driving superior rectus muscle contraction are needed to assess how their motor drive changes in response to sustained retrograde GDNF treatment. The potential role of GDNF on the control of muscle contractile strength is supported further by recent microarray, proteomics, and RNAseq studies that demonstrated decreased expression of GDNF in the EOM from adult strabismic subjects compared to age-matched controls [9, 10]. Dysregulation of GDNF in the EOM from strabismic subjects suggests it may play a role in either development or maintenance of the functional extraocular muscle imbalance in this disorder.

The sustained treatment of adult rabbit EOM with GDNF resulted in a surprising increase in mean myofiber cross-sectional area particularly in the global layer. The potential effect of GDNF on muscle size has not been well characterized in the literature. In general, the focus of the effects of increased GDNF, whether by exogenous or transgenic elevation, has been on motor neuron survival and improving peripheral nerve regeneration [32 33]. Studies have shown a correlation between elevated levels of GDNF and increased myofiber size after exercise [34]. Similarly, elevated levels of GDNF expression in regenerating muscles fibers in subjects with neuromuscular disease have also been described [35], as well as continued expression in extraocular muscles from subjects with amyotrophic lateral sclerosis [36]. While it has been shown that increased fiber size is not always proportional to increased force generation [37], the increased myofiber size coupled with the decrease in generated force was unexpected. The increased myofiber size was, however, coupled with decreased neuromuscular density, suggesting that innervation had not sufficiently adapted to the changes in mean myofiber area. In the present study, one month of GDNF treatment was performed. In a study using sustained IGF-1 treatment in rabbits where muscles were treated for 3 months, there was a distinct fluctuation in force measurements over time, with force generation increased at one month, decreased at two months, and increased even more dramatically at 3 months [3]. Using the same IGF-1 treatment paradigm in an adult strabismic monkey, eye alignment changed only 1–2° in the first two months, and only in the third month of sustained IGF-1 treatment did eye alignment significantly change, between 11–14° [7]. These findings suggest that the adaptation seen after only one month of sustained GDNF treatment would be further modulated after a longer duration of exposure.

In contrast to our previous study in infant monkeys, where 3 months of sustained IGF-1 treatment resulted in a coordinated increase in myofiber size and increase in neuromuscular junction size [6], no coordinated changes in neuromuscular junction size were seen in the present study. As with force generation, it may be that a longer duration of treatment is needed to allow adaptation of neuromuscular junctions to the altered myofiber cross-sectional areas. As neuromuscular junctions produce an all-or-none response in skeletal muscle, it may be that the efficacy at the average synapse was decreased [38, 39]. Other neurotrophic factors have been shown to modulate these properties [40, 41]. Further analysis is needed to determine the molecular changes at the neuromuscular junction in response to sustained GDNF treatment, but these are beyond the scope of the current study.

The increased percentage of myofibers positive for neonatal and developmental MyHC isoform expression levels in the global layer would be predicted to alter overall shortening velocity [20, 42, 43]. Similarly, an increase in slow tonic myofiber expression would be predicted to increase the time to complete muscle relaxation. Both are consistent with our results. These sorts of analyses are important, as the extraocular muscles show a complex pattern of co-expression in single muscle fibers as well as differences in expression patterns along the length of single muscle fibers [44, 45]. The changes in slow tonic MyHC expression mirror similar changes seen after BDNF treatment of rabbit superior rectus muscles [46], which also produced muscles with altered relaxation times. As the change was primarily in the global layer myofibers, it would have a greater impact on the overall muscle contractile characteristics, and is consistent with our current results.

The effect of changes to myofiber remodeling in EOM on overall contractile function is unknown. It is possible that increased myofiber remodeling may result in increased numbers of short myofibers. This would be predicted to result in greater lateral dissipation of muscle force, which is already significant in EOM [47–49]. It is interesting to note that experimental recession surgery in adult rabbits resulted in a significant increase in myonuclear addition as visualized by bromodeoxyuridine labeling [50]. In the short term, the increased rate of myonuclear addition could result in altered length/tension within the operated muscles, resulting in decreased force generation. Over time, the muscles ultimately are predicted to return to normal length/tension and normal force generation. In a recent study in adult strabismic monkeys, in the short term after surgery on the strabismic muscles improved eye alignment was seen. However, the motor neurons altered their neuronal drive over the next 6 month period post-surgery, resulting in regression of eye alignment over this period [51]. This was hypothesized to be due to both motor neuron and muscle adaptation over time.

Our preliminary data showed that 3 months of unilateral, sustained GDNF treatment in infant monkey medial and lateral rectus muscles resulted in the development of strabismus [16]. What is particularly noteworthy is that the pattern and timetable of change in angle of eye alignment differed between the three treated infant monkeys [16]. A similar picture was seen after unilateral IGF-1 treatment in infant monkeys [6]. Rabbits, unlike monkeys, are not binocular, with only a 9–18° overlap in visual field [52]. Data suggest that vertical extraocular muscles adapt differently compared to the horizontal muscles relative to coordinated changes in contralateral muscles [6, 8]. In the rabbit superior rectus, we saw significant changes to force and relaxation rates and duration in both GDNF treated and untreated muscles compared to naïve control superior rectus muscles. These differential results between horizontal and vertical rectus muscles may be related to their motor nerve innervation. The oculomotor nerve innervates both the superior and inferior rectus muscles, while the monkey studies examined the medial and lateral rectus muscles, with the medial rectus muscle innervated by the oculomotor nerve and the lateral rectus muscle innervated by the abducens nerve. This potentially makes the coordination of the adaptive changes after experimental perturbation more complex. While the presence of a fovea and binocularity in the non-human primate is likely to be a more critical factor when considering the effects of sustained neurotrophic factor delivery to these different muscles, the studies of the Das laboratory on adaptation of adult strabismic monkeys to strabismus surgery strongly support the direct effect of motor neuron drive on the short term maintenance and longer term regression of the angle of eye alignment [51, 53, 54].

The role of GDNF in altering myogenic stem cell division and/or differentiation in skeletal muscles is unclear. As GDNF is normally produced by skeletal muscles [35] and specifically by the extraocular muscles [12], its retention in mature EOM must be essential for normal oculomotor function. As GDNF levels were significantly reduced in human strabismic muscles [9, 10], it is not surprising that modulation of GDNF levels by exogenous sustained treatment would have measurable effects on muscle properties. The ability of 3 months of sustained release GDNF treatment to produce significant and maintained strabismus in infant monkeys [16] is further evidence for its potential impact in maintaining normal EOM function and eye alignment.

## Conclusions

One month of sustained delivery of GDNF to adult rabbit superior rectus muscles resulted in a significant muscle adaptation. GDNF altered muscle force generation relative to muscle size, increased myofiber mean cross-sectional areas in the global layer fibers, increased expression of neonatal, developmental, and slow tonic MyHC isoforms particularly in the global layer, and increased the numbers of Pitx2-positive myogenic precursor cells. These data suggest that in the absence of normal levels of GDNF, as seen in the strabismic human EOM [9, 10], sustained replacement of GDNF has the potential to improve eye alignment through its capacity to cause significant muscle adaptation over time.
